# ‘The Good Doctor’: the Making and Unmaking of the Physician Self in Contemporary South Africa

**DOI:** 10.1007/s10912-019-09572-y

**Published:** 2019-08-23

**Authors:** Michelle Pentecost, Thomas Cousins

**Affiliations:** 1 Department of Global Health and Social Medicine, King’s College London, London, UK; 2 School of African and Gender Studies, Anthropology and Linguistics, University of Cape Town, Cape Town, South Africa; 3 Institute for Social and Cultural Anthropology, University of Oxford, Oxford, UK; 4 Department of Sociology and Social Anthropology, Stellenbosch University, Stellenbosch, South Africa

**Keywords:** South Africa, Medical education, Care, Resilience, Endurance

## Abstract

In this article we examine the figure of the doctor in animated debates around public sector medicine in contemporary South Africa. The loss of health professionals from the South African public system is a key contributor to the present healthcare crisis. South African medical schools have revised curricula to engage trainee doctors with a broader set of social concerns, but the disjunctures between training, health systems failures, and a high disease burden call into question whether junior doctors are adequately prepared or whether conditions of care extend beyond medical training. A concern with 'resilience' suggests a correct ethical relation to a putative obligation to provide care in a struggling system. By examining the ways in which trainee doctors are expected to 'cope' with the demands of medical practice, to adopt the correct moral posture in relation to the urgency of care, and to enact a desirable ethical relation to the broader social and political context of medical practice, we examine the picture of humanist concern that animates the subjectivities and techniques of the self called for by this training, and advocate for *endurance* as an alternative framework for understanding the political and ethical relations between doctors, patients and health systems.

## Introduction

*'The first time I saw him I thought, he won't last.*

*I was sitting in the office in the late afternoon and he appeared suddenly in the doorway, carrying a suitcase in one hand and wearing plain clothes - jeans and a brown shirt -with his white coat on top. He looked young and lost and a bit bewildered, but that wasn't why I thought what I did. It was because of something else, something I could see in his face.'*

In the opening scene of Damon Galgut's 2003 novel *The Good Doctor*, the protagonist Frank Eloff, a weary, middle-aged clinician, is confronted with an interruption at the rural South African hospital where he works: Laurence Waters, a newly minted doctor has arrived to do his year of community service. Since 1998, community service has been compulsory for health sciences graduates in South Africa in the state sector as a means of redistributing human resources and improving health care in rural parts of the country, and as part of professional development (Reid et al. 2018). In Galgut's novel, the young doctor Laurence chooses a rural post because he wants to go to the most remote place possible; because he wants his work to 'mean something.' He finds a clinic where the sinks have been ripped from the walls, every last bit of metal stolen, and a few haggard doctors keep up a pretense of practice in the rundown clinic. The novel centers on the inexorable tension between Eloff's disenchantment and Waters' idealism, and offers a crystallized vision of the practice of medicine in post-apartheid South Africa and more broadly of life in this postcolony. Waters will learn the hard way about the harsh realities of practice in his rural posting, and in the end, Eloff's first impression is depressingly sound: he won't last.

South Africa, like many other sub Saharan countries, reports a critical shortage of doctors (South African Department of Health 2017). The World Health Organisation estimates that for every one thousand people, SA has 0.9 doctors. For comparison, Germany has 4.2, the United Kingdom (UK) has 2.8, Brazil has 2.1, Mozambique 0.07, and Zimbabwe 0.07 (World Health Organisation 2019). The South African health system comprises a private and a public sector. While it is difficult to obtain an accurate picture of the number of doctors working in South Africa and their distribution, recent findings by the South African Competitions Commission indicate a stark contrast in access to medical practitioners between these sectors: 1.75 practitioners per one thousand insured people in the private sector, versus 0.3 practitioners per one thousand people in the public sector (Competition Commission 2018). The public sector is divided into primary, district, secondary and tertiary levels, with referral required for specialist care. South Africa has eight medical schools that currently produce between twelve to thirteen hundred graduates per annum, which is considered to be an insufficient production rate for the country's requirements (Mahlathi and Dlamini 2017). Graduates have three years of compulsory practice in the public sector before they can practise independently, consisting of a two-year internship, during which time they are trainees, followed by a year of community service (Reid 2018). Allocations to hospitals for internship and community service are done by ballot, and approximately half of each cohort is allocated to rural facilities (Reid 2018). While a twenty year review of community service in South Africa shows that it has been a successful strategy for improving rural recruitment, it has not improved staff retention rates in rural areas (Reid 2018). After the community service year, graduates may find work in the public sector, begin specialist training, move to the private sector, or engage in work outside of clinical medicine. Of concern is the migration of doctors to other countries after their training, notably to the UK, Ireland, Australia, and Canada. According to a 2013 study, around half of South Africa's public sector health care workers are dissatisfied and intend to leave to work elsewhere **(**Blaauw et al. 2013). In 2011, the estimated loss of returns on state investment in medical education from this migration was 1.41 billion US dollars (Mills et al. 2011). While there is insufficient data on the mobility of South African medical doctors, poor retention rates threaten the viability of the planned implementation of a National Health Insurance package, which purportedly rests on the availability of doctors with a commitment to the collective benefits of this scheme (Mahlathi and Dlamini 2017). A fifteen-year longitudinal study of the compulsory community service scheme shows that while the majority of doctors did think that they had 'made a difference' and learned as professionals, around half felt that administrative and clinical support had been inadequate (Reid et al. 2018). In this fifteen-year cohort, expected rural retention remained constant with 15% of doctors indicating that they would continue to work in underserved and rural areas (Reid et al. 2018). In sum, the government currently trains too few doctors; of those who graduate, a majority will move into private practice, emigrate, or switch professions, and rural retention rates remain low.

In asking how this phenomenon is made sense of in public discourse in South Africa, we examine a range of sources, including novels, media coverage, blogs, articles, and books by doctors and students. What emerges from the various discursive constructions of the 'good doctor' (*pace* Damon Galgut) is a discussion of 'values' and 'value,' and the disjunctures between the physician-self and the social and professional contexts of the medical encounter.

This essay argues that if we are to understand the current crisis of health care in South Africa, we must attend to the apparent disjunctures between how the state, training institutions, medical students, and graduates themselves understand what it means to be a 'good doctor.' We argue that key to understanding the poor retention of doctors in the public sector is an understanding of what Michel Foucault referred to as 'techniques of the self' (1988) that are elicited in the making and unmaking of doctors in contemporary South Africa. Drawing on the various ways in which the figure of the physician appears in the media, novels, and medical students and graduates of their training, we explore distinct narratives of the 'good doctor' at work here, and the tensions and outcomes these produce. In particular we are concerned with the evolving interest in the concept of 'resilience' as a central tool for medical education and retention of doctors in South Africa and globally (Howe, Smajdor, and Stockl 2012; Eley, Wilkinson, and Cloninger 2013; Jenkins et al. 2015). Resilience in medical education literature is defined as the capacity to succeed in the face of adversity or stress (Howe, Smajdor, and Stockl 2012). Moving between institutional pedagogies, physician narratives, and the contexts of practice from which those narratives emerge, we suggest that the institutional shift to foster resilience in graduates rests on a particular set of values that privilege ideas of self-reliance and the pursuit of individual career advancement over commitment to patient wellbeing and attention to the ways in which suffering is structurally produced. To the extent that caregiving is possible in the context of a state-led attempt to maintain and expand a public health care system that appears overburdened and in chronic crisis, we offer *endurance* as a framework with positive political possibilities for breaking the impasse between state, university and student expectations of what it means to be a 'good doctor' in South Africa. Endurance is a key concept in Elizabeth Povinelli’s critique of what she calls ‘late liberalism’ that seeks to understand how specific ‘social forms of life’ persist – or ‘strive to persevere’ – under conditions that exclude their existence as anything other than liberal subjects responsible for their own fate (2011). That is, endurance is a means by which to understand the effort to constitute ‘social projects of the otherwise’ despite crushing conditions that exclude whole classes and categories of people. We explore below the tensions between resilience and endurance through a reflection on the available figures of the good doctor in contemporary South Africa.

## The figure of the 'good doctor'

In the classic text *The Illness Narratives: Suffering, Healing and the Human Condition* (1988), Arthur Kleinman offers a number of images with which to think about the experience of doctoring: *the wounded healer,* who has experienced personal suffering and wants to alleviate the suffering of others*; the burnt out carer,* ready to leave the profession*; the revolutionary,* politicised by the witnessing of structural violence; *the cynic,* who gets on with the job and ring-fences his role; *the Chinese healer*, who conceives of medicine as less a profession and more a 'wisdom of life'; and *the sensitive neophyte,* the young idealist who can see how his senior colleagues have hardened themselves to distress. While Kleinman is cautious that such caricatures are not representative of all healing experience, he provides them as a way into understanding that the medical encounter includes not one social being but two: patient *and* doctor.

Thinking with these images in the South African context, a number of such archetypes are at hand. Returning to Galgut's novel, Frank Eloff exemplifies the hardened cynic, while Lawrence Waters is the healer who needs to help (see also Malkki 2015). He has spent a year doing humanitarian work in the Sudan before arriving at the rural South African posting and wants to set up outreach clinics, despite lacking supplies. The good doctor is as much a discursive as a social figure, and several South African examples are at hand for sketching out this terrain.

The history of medicine in South Africa points to a number of archetypal forms or tropes that could potentially animate contemporary values in the profession. The first might be called 'the medical missionary,' best exemplified by the nineteenth-century physician and explorer, David Livingstone. The image of the medical missionary remains alive for young Christian doctors, inspiring a public commitment to rural medicine in South Africa. A second figure closely related to this is founded on a humanistic or secular concern for the poor and downtrodden, an image with its origins in the work of Sidney and Emily Kark, a husband-wife team who provided a cogent foundation for social medicine in rural South Africa in the 1940s and 1950s (Marks 1997). Frequently cited as global pioneers of primary health care, their example is followed by recent social movements such as the People's Health Movement. The political history of South Africa provides many examples of the revolutionary: Steve Biko, who abandoned his medical training for full time political involvement, was assassinated by the apartheid state in 1977; his partner, Mamphela Ramphele, who trained as a doctor and then went on to study anthropology, acted as vice-chancellor of the University of Cape Town, and then as managing director of the World Bank; James Moroka, an iconic political leader and rural general practitioner who was president of the ANC from 1949 to 1952 but later fell out with the organization over its Defiance Campaign of the 1950s, which he denounced as too radical. He was later to testify against his comrades at the trial that followed. Nelson Mandela excused him, saying, 'Dr Moroka's actions were motivated not by malice but by the desire to protect his medical practice' (Ncayiyana 2007). More recently, the figure of the revolutionary doctor has emerged most prominently in the protracted fight for access to antiretroviral treatment for HIV positive patients: Herman Reuter and Eric Goemaera, who spearheaded *Medicins sans Frontiers'* efforts to prove that a mass rollout was feasible, are perhaps the most well-known examples (Geffen 2010).

While such figures are available as role models for trainee doctors, there is one archetype that appears to dominate institutional and public imaginations of medicine in South Africa: that of the doctor who saves lives while advancing medicine and science through technology. Probably the most famous South African medical figure is pioneering the surgeon, Christiaan Barnard, who performed the world's first heart transplant in 1967 at Groote Schuur Hospital in Cape Town. A recent example of the pioneer clinician scientist is surgeon Andre van der Merwe, who performed the world's first penis transplant in 2016, a procedure that may assist young men who suffer penile loss following complications of ritual circumcision in South Africa (van der Merwe et al. 2017). The image of the pioneer remains the valued horizon of possibility for specialization and career advancement for South African doctors. It is the one animated in South African university prospectuses advertising medicine to prospective students: 'excellence,' 'science,' and 'specializing' are implicitly linked, while little mention is made of the proposed plan for National Health Insurance or the state expectation that the majority of graduates should continue work in the public sector or in primary care roles after graduation. Thus, there appear to be significant disjunctures between the popular images of medicine that institutions repeat when recruiting students; the humanist concern that animates institutional expressions of the 'graduate attributes' and values that are envisaged to produce 'ethical' and 'resilient' doctors; and the narratives of self which actually shape the experiences and choices of medical students and graduates.

By framing the question in terms of the figure of the doctor, we suggest that notions of vocation, political commitment, pastoral care, or even an entrepreneurial self are narratively constructed within particular genres of thought and writing, in addition to particular social, political, and professional histories of medicine, and that these manifest as competing values for trainee doctors.

## Competing values

These competing values are not openly articulated but also form the subtext for the tensions that arise between professionalism, a social ethic, and personal accomplishment. In a study of undergraduate medical students at the University of Cape Town, many students expressed their surprise at the heavy emphasis on primary health care in the undergraduate medical curriculum and the expectation that most graduates would go on to practice in primary care (Draper and Louw 2007). Another study at the University of Pretoria assessed students' perceptions of practising 'professionalism' in South Africa, defined as including values such as respect for patient autonomy, social justice, professional competence, confidentiality, quality of care, access to care, and commitment to scientific knowledge (van Rooyen 2004). Two thirds of students surveyed agreed that 'The physician is in the unenviable position of having to balance professionalism and moral obligation with the need for self-fulfillment and personal success' (29). In van Rooyen's study, some cited these values as 'utopian,' possible in 'an ideal, sophisticated society,' and highlighted the socioeconomic and political barriers to practising such values (29). Only a quarter of the students agreed that high quality care should be available to all and that such obstacles should be overcome (van Rooyen 2004).

The South African example resonates with work done elsewhere on competing values that animate medical students' concerns. In the United States, Kasper and colleagues outline three critiques that medical students at Harvard Medical School present when discussing the boundaries of their clinical mandate. The 'antisocial' critique argues that clinicians should not be called upon to do the work of 'social workers, economists and politicians' (Kasper et al. 2016). The 'pragmatic' critique argues that any knowledge outside of that which will ensure success as a clinician (technical knowledge) is irrelevant; and the 'local' critique argues that there is no need to learn about health issues elsewhere if one will work solely in the United States. The antisocial and pragmatic attitudes are equally prevalent in the South African medical classroom, while the local critique sees an interesting North/South inversion: for South African medical students eager to practise in the UK, Canada, and Australia (the major international destinations for South African doctors), the need for technical excellence is amplified by the desire to be seen as internationally competent. The humanitarian crises that might animate the American imagination of global health in Africa are likewise 'out there' in Africa for the South African medical student who must train in situations of ordinary crisis (Pentecost and Cousins 2018).

The students' expectations in the US and SA examples given above show that today's students are still strongly attached to the image and identity of a doctor as a 'scientist' (see also Gerber 2016). Their their work, once they are practising, cannot be disentangled from the social, it must entail involving oneself in the messiness of people's lives, it does not conform to neat protocols, and the pharmacological mechanism of the antiretroviral is as important as the patient's access to food in order for her to be able to take it is. These realities are an affront to the assumption that doctors are figures of authority who will dispense truth based on science and perform noble life-saving procedures.

Thus, students' expectations and espoused values might be echoed by university prospectuses but do not align with institutional or state expectations for medical graduates. Post-graduation, these disjunctures between the moral life of the student—the lived values that may have led her/him to choose medicine—and the local worlds in which graduates find themselves manifest in a number of ways that have received much attention in the South African media.

In 2017, the media covered a protracted fight between the Health Minister and doctors not yet placed for their postgraduate internships. The graduating doctors claimed that they had not been allocated posts and were without employment due to the poor administration of the Health Department. The Minister argued that these doctors had declined to apply for the remaining posts in the country—concentrated in rural areas. While the government argued that it was not compelled to offer graduates a choice about where they complete their internship, graduates considered the system unfair and wanted to be placed near to family (often in urban centers such as Cape Town or Johannesburg) and in facilities with amenities. This impasse is perhaps the most concrete expression of the distance between state and graduate expectations. Some doctors have even harnessed a 'human rights' discourse to protest their working conditions; the most publicized case is Dr Miguel Desroches, who approached the Constitutional Court when he was denied a community service post in the Western Cape, one of the country's more affluent provinces (Bateman 2014). Desroches called for the Court to rule the Community Service Regulations in Section 24A of the Health Professions Act (1974)[1] as unconstitutional. The judges dismissed the application.

More prominently, the problem of burn-out has repeated itself across the media, doctors' blogs, publications, and a 2016 feature-length documentary entitled, *Doc-u-mentally* that recorded the lives of doctors on call for shifts of up to thirty hours, which remains the legal working limit for doctors in South Africa despite recent protests and concern about the safety of long working hours (Wahl 2016). The 'super-hero' trope here finds dangerous expression in the state and public expectation that doctors can and should work for such long periods. For those who genuinely espouse values of caregiving and healing, these values are incompatible with a dysfunctional system that endangers healthcare workers' lives—the death of an intern in early 2017 who fell asleep at the wheel en route home after a long shift being a tragic example. The feelings of personal failure that such a discourse engenders is summed up by Dr Maria Phalime in her 2012 memoir, *Postmortem - The Doctor Who Walked Away.* Phalime writes: 'This is my story and the stories of other doctors who chose to walk away … Ours is a private anguish filled with the niggling suspicion that we should have been stronger, more committed, more able to handle the daily realities of practicing medicine in South Africa.' In moving prose, it is often in these contexts that a language of 'resilience,' indexing an autonomous, liberal subject who must develop inner, personal resources is painfully expressed as a personal failure of resolve or commitment to a profession or to an overwhelming social context. What if we read these expressions sympathetically in a different direction, one more attuned to the way in which the self is produced by and through socially mediated discourse and practice? It is here that we offer the notion of 'endurance' as an alternative way to think about value and virtue in relation to the figure of the doctor in order to bring to the fore a set of social and political questions that challenge the training and employment of doctors in public service.

## Doctoring the self

Much work in medical anthropology and related disciplines has urged that *'patienthood is a social state*, rather than simply a biological one' (Eisenberg and Kleinman 1981, 12 emphasis in original). However, less attention has been paid to what it means to be a doctor, to how one becomes a doctor, and the doctor's role in contemporary clinical practice. If the 'decision to become a patient' is a social one (Eisenberg and Kleinman 1981, 12), so too is the decision to become a doctor. The decision to become a patient is 'almost always with the advice of others' (ibid 13) and made against a 'background of values which vary with social class and culture' (ibid 13). The same can be said for the decision to become a doctor. We thus might speak in terms of *physicianhood*, to get at the ways in which the subject position of becoming and being a doctor is shaped by social, cultural, political or economic processes, and subject to local moral worlds and how those worlds shape one's own sense of ethical conduct and practice. Kleinman and Hanna argue that the flow of experience, namely of words, movement and emotions between us, is inherently moral:

Why so? Because living our lives is about animating and enacting values. We are constantly experiencing, negotiating, defending and just living values. Those lived values are the things that are personally and collectively at stake for us: for example, status, reputation, resources, connections, religious and cultural practices, and so on. Moral experience is the flow of things at stake in local worlds. Our own moral life may be consistent with or in conflict with our local worlds of experience. (2008, 293)

While we find useful the appeal to consider 'values' and contextualize these in 'local moral worlds,' we are not certain that these values always travel so easily and if so, by what means. What is clear is that 'lived values' take very different forms in different places and times and that we need to pay attention to what happens when 'local worlds' are shot through with fragmented or partial forms of citizenship, or staked on complex forms of care and authority. In the South African context, understanding the doctor as a social figure is staked on the possibility of being attuned to the historical and political contexts that shape medical training and practice. Kleinman and Hanna argue that clinicians are moving 'further from the intimate engagement with another's suffering that is inherent in the labor of taking care of another human' (2008, 288), and they advocate for a return to 'caregiving' in the medical profession. While this is one part of how we might reconfigure the figure of the doctor, we suggest that 'the logic of care' (Mol 2008) is only part of what is required. Part of that logic of care must also include an *attuning* to the particular historical, political and social configuration in which doctor and patient meet in order that the physician might hold and bear this set of relations and the suffering it engenders, even in the face of only being able to address the very immediate concern of the patient, which we have elsewhere termed 'relatedness' (Pentecost et al. 2018). In a 2011 *Lancet* editorial, Kleinman argues that medical education must include some means by which students are taught to recognize their own hidden values and the images that animate their practice - no less an exercise in 'the moral building of the clinician as a fully developed human being' (805). We suggest going one step further by considering this in the distinct sociopolitical context of post-apartheid South Africa. There is ample anthropological literature on healing to understand how the clinical and the social are braided together in this particular context (Last and Chavanduka 1986; Feierman 1985; Janzen 1992; Livingstone 2012; Packard 1989).

We return to the beginning of this debate in our field to think with Michael Taussig's response to the initial turn to a 'medical anthropology' that seeks to understand the clinical encounter. In 1980, he argued that 'in denying the human relations embodied in signs, symptoms, and therapy, we mystify those relations and also reproduce a political ideology in the guise of a science of physical things' (3). He argues that it is 'in therapy' that 'socially engendered categories' are ratified: 'In any society, the relationship between doctor and patient is more than a technical one. It is very much a social interaction, which can reinforce the culture's basic premises in a most powerful manner' (4). Drawing on the work of Victor Turner and Claude Levi-Strauss, Taussig reminds us that the patient is a medium through which societal problems might be adapted and attended to, and this is the challenge of the doctor: 'charged with the emotional load of suffering and of abnormality, sickness sets forth a challenge to the complacent and everyday acceptance of conventional structures of meaning… It is not the cultural construction of clinical reality that is here at issue, but the clinical construction and reconstruction of a commoditized reality that is at stake. Until that is recognized and acted upon, humanistic medicine is a contradiction of terms' (13).

Taussig points here to an important requirement for the doctor: that she must bear the burden of the contradictions of the society in which she works. Sickness—her daily bread—will challenge daily the 'complacent and everyday acceptance of conventional structures of meaning': in contemporary South Africa, the everyday complacency that renders parts of cities, whole populations, invisible. As such, the doctor is confronted daily with a politics of recognition (Povinelli 2011) 'charged with the emotional load of suffering and of abnormality', and constructs clinically a means by which the social order can stand, and the conflict can be abated. We concur with Taussig that 'until that is recognized and acted upon, humanistic medicine is a contradiction of terms' (1980, 13). Apartheid produced a distinct form of suffering and abnormality that implicated and impacted on doctors and the practice of medicine in particular ways, as many scholars have shown (e.g. Coovadia et al. 2009; Dubow 2014; Packard 1989). These modes of suffering continue in the post-Apartheid era, and doctors continue to be challenged to respond to that suffering.

In his 1985 article, 'Struggles for Control: The Social Roots of Health and Healing in Modern Africa,' Steven Feierman shows that 'values are not narrowly restricted to the sphere of medicine – they pervade society as a whole. The person who controls therapy serves as a conduit transmitting general social values, but is also capable of reshaping and reinterpreting those values in the healing process' (75). How then, to practice medicine in contemporary South Africa and settings like it? It is no less true today that, in Feierman's assessment, 'There is invariably a contradiction between the unequal distribution of social costs and the professional ethic of public service' (115). The doctor is called to acknowledge this and bear it, not merely in principle but in the ordinary practice of healing and curing in everyday clinical settings.

While medical students today might not recognize the names of James Moroka, Sidney Kark, Christiaan Barnard, or Herman Reuter, they would certainly recognize the various figures of the hero, the revolutionary, the surgeon specialist, and the rural doctor. As Damon Galgut demonstrates in literary form, and the *Doc-umentally* documentary in visual form, the passion of the primary care doctor or the public sector specialist stands opposed to the self-interest of the careerist who must specialize in order to escape the horror of the junior doctor in public service. When young doctors say that after a thirty-hour shift, they no longer have the physical capacity to render care, despite their undiminished passion for their work, there are several intersecting concerns captured in their expression.

First, the doctor rendered as technician operating protocols and machines, his body a machine depleted of physical strength, stands opposed to the image of the healer whose art is rendered via the magic of hands-on care. The doctor-as-machine imaginary poses a regulatory question for the state and its concern for civil servants and citizens, given the lack of labor regulations protecting workplace health and safety concerning doctors' working hours. Second, the notion of forced 'voluntary labor' as a 'slave to the state,' as doctors such as Desroches have framed it (Bateman 2014), offends the premise that doctors' commitments to public service is founded on an ethical obligation to the body politic. Third, underlying the shortages of personnel is a political economy of doctors spread between private and public practice, which pits social values against private choice. It is here that Michel Foucault's analysis of the neoliberal subject who must become an entrepreneur of the self (2008) resonates powerfully. Career advancement, success, happiness, income, appear in most public debates around the departure of young doctors from the public sector in South Africa. Thus, care versus choice, on the one hand, and resilience versus preservation of the self, on the other, presents itself as a tangled knot—a double-bind drawing together patients and their doctors (see also Pentecost 2018).

## Enduring

We suggest that the concept of *endurance* might prove useful in thinking about how practitioners can engage with the tensions we have described above without the individual responsibility that the concept of resilience has frequently suggested. Might we think rather of *enduring* as a modality of clinical encounter that draws physician and patient together in particular political, social, economic contexts? Here we are thinking with Elizabeth Povinelli who, from another direction, has suggested 'endurance' as a political/existential modality through which those who are made superfluous by capitalism attach ambivalently to life (2011). Certainly, the current inequity in the distribution of medical and public health care; of the burden of disease and pathology; and of life outcomes in South Africa suggests a 'distribution of hope and harm,' as Povinelli puts it, premised on a hyphenated private-public arrangement of capacity and concern. Endurance in this sense is not about an abstract commitment to a value – for example, one should be concerned for the poor – but a concrete engagement with an actual set of arrangements that takes the social projects of those excluded by liberal politics as the starting point for a building of more just worlds. Thus, patients and doctors together might work from a recognition of the effort of endurance, that is the effort required to persist, to stay alive stubbornly (Povinelli 2018), and with available, limited materials (no gloves, limited drugs, hunger, bodily exhaustion), on the side of life.

## Conclusion

In the South African state's current attempt to institute universal health care coverage, it assumes a solution to the problem of the unavailability of doctors in the public sector. Central to this solution is an appeal to a social ethic to which training doctors are imagined to subscribe. We have focused on the South African example, but these questions are relevant to clinical contexts globally. As anthropologists we know that each locality will come with its own ecology of values and practice; this offering from the South African setting will certainly inflect in both similar and different ways elsewhere. Despite those contextual differences, the concept of endurance allows us to draw together an acknowledgement of the dire socio-economic conditions under which many structurally excluded people in South Africa exist, and the demands placed on training and practising doctors in the public sector. Endurance invites a different relationship of the self to the various figures of the doctor at hand, and places that relationship in a socially and historically embedded context.
